# Circadian clock dysregulation: a potential mechanism of depression in obstructive sleep apnea patients

**DOI:** 10.1038/s41398-024-03134-0

**Published:** 2024-10-07

**Authors:** Agata Gabryelska, Szymon Turkiewicz, Piotr Kaczmarski, Adrian Gajewski, Piotr Białasiewicz, Dominik Strzelecki, Maciej Chałubiński, Marcin Sochal

**Affiliations:** 1 Department of Sleep Medicine and Metabolic Disorder, Medical University of Lodz, Lodz, Poland; 2 Department of Immunology and Allergy, Medical University of Lodz, Lodz, Poland; 3 Department of Affective and Psychotic Disorders, Medical University of Lodz, Lodz, Poland

**Keywords:** Clinical genetics, Psychiatric disorders

## Abstract

Obstructive sleep apnea (OSA) is characterized by co-occurrence with affective disorders. Our study aims to investigate the association of circadian clock gene expressions, and the presence and severity of depressive symptoms in OSA patients. The study included 184 individuals, who underwent polysomnography (PSG) and had their peripheral blood collected in the evening before and the morning after the PSG. Patients were divided into two groups: the OSA (apnea-hypopnea index (AHI) > 5) and the control group (AHI < 5). RNA was extracted from peripheral blood leukocytes. Expression levels of the selected genes (*BMAL1*, *CLOCK*, *PER1*, *CRY1*, *NPAS2*, and *NR1D1*) were assessed by qRT-PCR. Questionnaire data was collected in the morning (including the Insomnia Severity Index (ISI), Epworth Sleepiness Scale (ESS), Chronotype Questionnaire (CQ), and Montgomery–Åsberg Depression Rating Scale (MADRS)). The expression of all examined circadian clock genes in OSA patients was upregulated in the morning compared to the evening (except *NPAS2*). No differences were observed between OSA and control groups at either time point. Additionally, there was a positive correlation between the severity of depressive symptoms (assessed with MADRS) and morning expression of circadian genes in the group of OSA patients. Finally, in multivariable linear regression, ISI score (*B* = 0.750, *p* < 0.001), AM score of CQ (*B* = 0.416, *p* = 0.007), and morning *PER1* gene expression (*B* = 4.310, *p* = 0.042) were found to be predictive factors for greater severity of depression symptoms in OSA patients. Dysregulated circadian clock gene expression in OSA patients is linked to depressive symptom severity, suggesting circadian disruption may underlie affective symptoms in OSA.

## Introduction

Circadian rhythms are endogenous regulators responsible for adapting human physiological and behavioral processes to changes during the day/night cycle. They are driven by an intracellular timing system that relies on the transcriptional regulation of gene networks [1, 2]. The primary genetic determinants responsible for regulating the presence and function of circadian rhythms are known as circadian clock genes. Rhythmic transcription of these genes regulates the expression of numerous other genes through feedback loops [2, 3]. This autonomous intracellular regulatory mechanism of circadian rhythm controls the switch in metabolic function and cell activity during the 24-hour cycle, partly independent of the central master clock in the suprachiasmatic nucleus [1, 2]. Up to date, several established circadian genes have been identified to regulate intracellular rhythms, they are often referred to as “clock genes” [4]. The most important feedback loop that drives intracellular circadian rhythms is the interaction between activators: circadian locomotor output cycles kaput (*CLOCK*)/ brain and muscle ARNT-Like 1 (*BMAL1*) and repressors: period (*PER*), cryptochrome (*CRY*). CLOCK and BMAL1 proteins form a heterodimer responsible for the transcription of many genes including *PERs*, *CRYs*, RAR related orphan receptor α (*RORα)*, and nuclear receptor subfamily 1 group D member 1 *(NR1D1)* [5]. PER-CRY complex after the phosphorylation acts as a repressor of CLOCK:BMAL1 dependent transcription. In addition to the primary feedback loop, other genes contribute to the regulation of circadian rhythms. NR1D1 protein binds to receptor-related orphan receptor response elements inhibiting the expression of the *BMAL1* and *CLOCK* genes [5]. The neuronal PAS domain protein 2 (*NPAS2*) gene has the capacity to form a dimer with BMAL1, thereby replacing CLOCK and fulfilling its role [5]. Rhythmic changes in repressors’ levels influence the cyclic transcription of circadian clock-controlled genes and therefore regulate intracellular metabolism and overall functioning of numerous systems. The cyclic transcription of clock genes is recognized to modulate metabolic processes such as lipid, glucose, and redox regulation, while also influencing the functionality of the endocrine, cardiovascular, and immune systems, in addition to governing sleep, behavior, and body temperature [1, 5–8].

Obstructive sleep apnea (OSA) is a common chronic sleep respiratory disorder with an estimated prevalence in the general population ranging from 9% to 38% [9]. The main pathological factors of OSA - sleep fragmentation and chronic intermittent hypoxia, reduce time and quality of sleep and therefore influence the physiological circadian clock mechanisms in OSA patients. There are numerous literature reports that hypoxia influences the circadian clock [10, 11]. Recently it has been described that hypoxia-inducible factor 1 (HIF1) a heterodimeric transcription factor that belongs to the basic helix-loop-helix PER-ARNT-SIM (bHLH-PAS) factor family (the same as BMAL1 and CLOCK), especially its α subunit is chronically upregulated in OSA [12]. Under hypoxic conditions in OSA, the α subunit of HIF-1 becomes stabilized and dimerizes with the β subunit, resulting in the formation of an active transcription factor. The exact connection between hypoxia and circadian rhythm dysregulation is not clear, although some hypotheses suggest that upregulated HIF-1 in OSA may target E-box-like hypoxia response elements (HRE) in promotors of *PERs*, *CRYs*, and *CLOCK* genes and therefore influence the expression of circadian rhythm genes [13].

Affective disorders are one of the most common comorbidities in OSA patients. It has been described that the prevalence of major depressive disorder (MDD) in OSA patients oscillates around 17% [14]. Many OSA and MDD patients present similar symptoms including excessive daytime sleepiness, anxiety, restlessness, fatigue, poor concentration, and poor sleep quality [14]. The pathology of OSA including sleep fragmentation, sleep architecture disruption, and intermittent hypoxia may contribute to the development of depressive symptoms [14]. Apart from that, many authors suggest that disturbances in the circadian clock, which is characteristic form both diseases might be a reason for their common coexistence [1, 15]. There are numerous reports that interference in the circadian clock e.g. shift work, or jet lag contributes to the development of affective disorders [16]. Alterations in the circadian clock in individuals with depression are frequently reflected by disruptions in sleep architecture. The desynchronization of circadian clock gene expression and therefore loss of circadian rhythms is one pathophysiological pathway leading to depression [17]. OSA and related molecular circadian clock disruption could lead to the development of depressive symptoms.

Thus, the study aimed to investigate the association of circadian rhythm through circadian clock gene expressions and questionnaire data with depression presence and severity in OSA patients as well as search for possible predictive factors (connected with circadian rhythm) for depression.

## Patients and methods

### Sample

A total of 184 individuals were recruited from the Sleep and Respiratory Disorders Centre in Lodz (Poland). All participants qualified and underwent a diagnostic polysomnography (PSG) examination. Based on the results the apnea-hypopnea index (AHI) was calculated and used to assign participants into two groups: the obstructive sleep apnea (OSA) group (AHI > 5) and the control group (AHI < 5). The inclusion criteria for the study comprised of age between 18 and 75 years and body-mass index (BMI) between 20 and 45 kg/m^2^. While following criteria were the basis for the exclusion of participants from the study: inflammatory diseases, chronic respiratory diseases, any infection within one month of blood collection, diagnosis of cancer in medical history, major neurological disorders, diagnosed psychiatric disorders, with insomnia and depression during the treatment period and taking medications affecting sleep (e.g., benzodiazepines and melatonin). All participants provided written informed consent to participate in the study. The Ethics Committee of the Medical University of Lodz approved the study (RNN/432/18/KE). All methods were performed in accordance with the relevant guidelines and regulations.

### Polysomnography

After the arrival at the sleep laboratory, the participants were subjected to a physical examination, which included measuring their height, weight, heart rate, and blood pressure, which was followed by nocturnal polysomnography (PSG) recording. The following channels were used during PSG: electroencephalography (EEG) for monitoring brain activity, electromyography (EMG) of the chin muscles and anterior tibialis for assessing muscle activity, electrooculography (EOG) for recording eye movements, thermistor gauge for measuring oronasal airflow, snoring recordings, body position tracking to monitor sleep position, piezoelectric gauges for measuring respiratory movements of the chest and abdomen, unipolar electrocardiogram (ECG) for monitoring heart activity, and hemoglobin oxygen saturation (SpO2) (Alice 6, Phillips-Respironics). Sleep stages were scored based on the 30-second epoch standard according to the guidelines of the American Academy of Sleep Medicine (AASM) [18]. Apnea was defined as a reduction in airflow to less than 10% of baseline for at least 10 seconds, while hypopnea was characterized by a minimum 30% decrease in airflow for at least 10 seconds, accompanied by a SpO_2_ decrease of over 3% or arousal. The events present during sleep were scored according to AASM guidelines [18].

### Material collection and assessment of mRNA level

Blood samples were obtained from participants in the evening before and the morning following a PSG examination using tubes with EDTA. The initial collection occurred 15 minutes before the lights out (around 9:00 PM), and the second collection occurred within 10 minutes after awakening (around 6:00 AM).

RNA was extracted from peripheral blood leukocytes (PBLs) using TRIzol (Invitrogen, Thermo Fisher Scientific Inc., California, United States). The quality of the isolated RNA was evaluated with the high-throughput Nanodrop Colibri Microvolume Spectrometer (Berthold Technologies, Zug, Switzerland) and assessed through RNA Integrity Number and concentration measurement. The isolated RNA underwent reverse transcription into cDNA following the manufacturer’s instructions (SuperScript IV First-Strand Synthesis System, Thermo Fisher Scientific Inc., California, United States), involving three steps, including primer annealing at 60 °C for 60 seconds. Quantitative real-time polymerase chain reaction (qRT-PCR) was used to determine the expression levels of the selected genes (*BMAL1*, *CLOCK*, *PER1*, *CRY1*, *NPAS2*, and *NR1D1*). The qRT-PCR mixture included nuclease-free water, a master mix, cDNA, gene-specific probes targeting the examined genes, and a reference gene (*β-actin*). Three qRT-PCR reactions were conducted for each sample and the reference gene. The cycle threshold (CT) was determined for each sample and the reference gene, and the difference in CT values (∆Ct) was calculated to analyze mRNA expression levels. The mRNA expression level was determined using the equation: 2^−∆Ct^ and presented after multiplication by 100.

### Questionnaires

In the morning after the PSG examination participants completed the following questionnaires Insomnia Severity Index (ISI) and Epworth Sleepiness Scale (ESS), while the Montgomery–Åsberg Depression Rating Scale (MADRS) was filled out by a qualified physician.

#### ISI

The Insomnia Severity Index (ISI) is a self-report questionnaire consisting of seven questions that assess the severity of insomnia symptoms [19]. Participants rate their sleep quality over the past month on a scale of 0 (no difficulty) to 5 (extreme difficulty). Total scores range from 0 to 28, with higher scores indicating more severe insomnia. Scores of 0 to 7 indicate no clinically significant insomnia; 8 to 14 suggest borderline insomnia; 15 to 21 indicate moderate insomnia; and 22 to 28 indicate severe insomnia [20].

#### ESS

The Epworth Sleepiness Scale (ESS) is a brief, self-administered questionnaire that is used to assess daytime sleepiness [21]. It consists of eight questions that ask respondents how likely they are to doze off or fall asleep in eight different situations. Participants rate each situation on a scale of 0 (no chance of dozing off) to 3 (high chance of dozing off). The total score for the ESS ranges from 0 to 24, with higher scores indicating more severe daytime sleepiness [21].

#### CQ

The Chronotype Questionnaire (CQ) is a tool used to evaluate a person’s sleep-wake preference in two dimensions: morningness-eveningness (ME) and subjective amplitude (AM) (each dimension including 8 items). Higher ME scores indicate a stronger preference for morning activities, while higher subjective AM scores indicate a greater amplitude [22]. The CQ consists of eight items for ME and eight items for subjective AM, with scores ranging from 8 to 40 for each dimension [22].

#### MADRS

The Montgomery- Åsberg Depression Rating Scale (MADRS) is a 10-item scale that assesses the severity of depressive symptoms [23]. Physician rates individuals’ symptoms on a scale of 0 to 6. The MADRS yields a total score that ranges from 0 to 60, with higher scores indicating more severe depression. Scores of 0 to 6 are indicative of no depression, 7 to 19 suggest mild depression, 20 to 34 indicate moderate depression, and 35 to 60 suggest severe depression [23].

### Statistical Analysis

SPSS 28.0 (IBM, Armonk, NY, USA) was used for statistical analysis. The level of statistical significance was set at *p* < 0.05. To assess the distribution of variables, the Shapiro–Wilk test was employed. Parameters exhibiting a normal distribution were compared using independent Student’s *t* tests, while non-normally distributed parameters were analyzed using Wilcoxon and Mann–Whitney *U* tests. Nominal variables were evaluated using chi-square tests. Spearman’s rank correlation was used to examine correlations. Multivariable linear regression with a stepwise procedure was applied to investigate the predictive factors of the MADRS score.

## Results

54 individuals were included in the control group, while the OSA group comprised 130 participants with a median AHI of 25.8 events/hour. Out of the evaluated questionnaires OSA group had lower scores on the ME dimension of CQ (*p* = 0.002) and MADRS (*p* = 0.038). The baseline demographics, PSG, and questionnaire characteristics of study groups are presented in Table 1. In the OSA group, 38 individuals had a MADRS score between 0–6 (no depression), while 75, 15, and 2 participants met the criteria for mild (MADRS score between 7–19), moderate (MADRS score between 20–34), and severe (MADRS score of 35 and over) depression respectively.

**Table 1:** Baseline demographic, polysomnography, and questionnaire characteristics of study groups.

Parameter	Control group (*n* = 54)	OSA group (*n* = 130)	*p* value
Demographics			
Age [years old]	44.8 12.4	53.9 11.7	**<0.001**
Sex (male [*n*])	36 (66.7%)	108 (83.1%)	**0.005**
BMI [kg/m^2^]	28.4 5.9	32.2 6.0	**<0.001**
PSG parameters			
Sleep efficiency [%]	84.2 (73.6–90.4)	82.7 (74.4–90.0)	0.970
Sleep onset latency [min]	23.0 (10.8–35.8)	19.8 (10.1–33.0)	0.203
Sleep maintenance efficiency [%]	89.7 (82.8–96.8)	89.5 (82.3–93.8)	0.399
REM sleep latency [min]	103.0 (78.3–142.6)	90.5 (67.5–152.3)	0.789
TST [h]	6.2 (5.5–7.2)	6.3 (5.4–7.0)	0.813
REM [h]	1.3 (0.9–1.7)	1.2 (0.8–1.7)	0.642
N1 nREM [h]	1.0 (0.5–1.4)	1.8 (1.1–2.5)	**<0.001**
N2 nREM [h]	2.8 (2.2–3.4)	2.1 (1.4–2.8)	**<0.001**
N3 nREM [h]	1.0 (0.7–1.6)	0.9 (0.4–1.3)	0.176
nREM [h]	4.8 (4.4–5.6)	4.9 (4.3–5.5)	0.990
Arousal index [events/h]	9.3 (5.0–12.9)	17.5 (10.9–25.2)	**<0.001**
AHI [events/h]	1.7 (1.0–3.1)	25.8. (11.7–46.4)	**<0.001**
AHI in REM [events/h]	2.5 (0.0–4.9)	26.8 (9.5–47.2)	**<0.001**
AHI in nREM [events/h]	1.3 (0.7–2.7)	20.9 (10.0–43.2)	**<0.001**
Total number of desaturations	10.5 (6.0–19.8)	146.0 (65.5–298.5)	**<0.001**
Desaturation index [events/h]	2.0 (1.0–3.0)	26.0 (11.2–49.5)	**<0.001**
Basal SpO_2_ [%]	94.1 (93.0–95.1)	92.5 (91.0–94.0)	**<0.001**
Mean SpO_2_ during desaturations [%]	90.9 (88.9–92.6)	88.3 (86.0–90.0)	0.893
Minimum SpO_2_ [%]	88.6 (85.7–90.9)	79.9 (71.0–84.0)	**<0.001**
Questionnaires			
ESS score	9.0 (5.5–11.0)	8.0 (5.0–12.0)	0.978
ISI score	14.0 (10.8–18.0)	13.0 (9.0–17.0)	0.199
ME score of CQ	23.0 (18.0–27.0)	20.0 (17.0–23.0)	**0.002**
AM score of CQ	23.0 (18.0–26.0)	21.0 (18.0–25.0)	0.216
MADRS score	14.0 (7.0–20.3)	11.0 (6.0–16.0)	**0.032**

*AHI* apnea-hypopnea index, *AM* subjective amplitude, *BMI* body mass index, *CQ* Chronotype Questionnaire, *ESS* Epworth Sleepiness Scale, *ISI* Insomnia Severity Index, *MADRS* Montgomery-Åsberg Depression Rating Scale, *ME* morningness-eveningness, *nREM* non-rapid eye movement, *OSA* obstructive sleep apnea, *REM* rapid eye movement, *SpO*_*2*_ oxygen saturation, *TST* total sleep time.

Bold values in the tables mark statistically significant data.

No significant differences in gene expression of the analyzed circadian clock genes were detected during either the morning or evening time points (*p* > 0.05). In the control group, higher gene expressions in the morning compared to the evening were obtained for *CRY1* (*p* = 0.003), *PER1* (*p* = 0.001), and *NR1D1* (*p* = 0.005). Among OSA participants all circadian clocks but *NPAS2* (*p* = 0.094) gene expressions were higher in the morning. All comparisons of circadian clock genes are presented in Table 2.

**Table 2:** Comparisons of circadian clock genes between control and OSA groups.

Gene expression		Control group	OSA group	*p* value control group vs. OSA group	*p* value evening vs. morning control group	*p* value evening vs. morning OSA group
Evening	*BMAL1*	6.73 (2.43–16.42)	6.64 (2.52–13.08)	0.855	0.082	**0.035**
Morning		10.70 (3.50–25.69)	8.02 (3.35–18.09)	0.191		
Evening	*CLOCK*	2.44 (1.58–6.98)	2.43 (1.20–4.44)	0.181	0.081	**0.007**
Morning		5.36 (2.49–12.47)	4.42 (2.09–10.43)	0.604		
Evening	*CRY1*	1.60 (1.10–7.03)	2.42 (0.95–4.76)	0.875	**0.003**	**0.003**
Morning		4.97 (1.63–12.81)	3.68 (1.80–9.03)	0.349		
Evening	*PER1*	4.18 (1.74–13.22)	5.36 (1.19–13.69)	0.910	**0.001**	**<0.001**
Morning		20.34 (8.09–38.67)	12.75 (3.57–27.97)	0.062		
Evening	*NPAS2*	0.65 (0.21–1.39)	0.48 (0.15–1.29)	0.292	0.683	0.094
Morning		0.93 (0.25–2.08)	0.79 (0.28–2.09)	0.857		
Evening	*NR1D1*	3.78 (1.65–10.07)	3.04 (1.32–6.24)	0.323	**0.005**	**<0.001**
Morning		8.02 (4.53–24.88)	7.57 (3.15–18.35)	0.469		

*BMAL1* brain and muscle ARNT-like 1, *CLOCK* circadian locomotor output cycles kaput, *CRY1* cryptochrome 1, *NPAS2* neuronal PAS domain 2, *NR1D1* nuclear receptor subfamily 1 group D member 1, *OSA* obstructive sleep apnea, *PER1* period 1.

Bold values in the tables mark statistically significant data.

In the OSA group, positive correlations were observed between all morning circadian clock gene expressions and the MADRS score, while none of the evening gene expressions were associated with the MADRS score (*p* > 0.05). In the control group, evening expression of the circadian clock gene NPAS2 was the only one found to correlate with the MADRS score (*R* = 0.294, *p* = 0.042). All evaluated correlations between MADRS score and gene expressions, demographics, questionnaire, and PSG variables are presented in Table 3.

**Table 3:** Evaluated correlations between MADRS score and demographic, questionnaire, gene expression, and polysomnographic variables.

	Parameter		MADRS score	
			*R*	*p* value
Demographics	Age		−0.032	0.719
	BMI		**0.183**	**0.041**
Questionnaires	ME score of CQ		**0.332**	**<0.001**
	AM score of CQ		**0.557**	**<0.001**
	ISI score		**0.611**	**<0.001**
	ESS score		**0.175**	**0.047**
Gene expression	Evening	*BMAL1*	−0.047	0.624
	Morning		**0.247**	**0.011**
	Evening	*CLOCK*	0.040	0.682
	Morning		**0.259**	**0.009**
	Evening	*CRY1*	−0.002	0.986
	Morning		**0.243**	**0.014**
	Evening	*PER1*	−0.009	0.926
	Morning		**0.293**	**0.003**
	Evening	*NPAS2*	−0.076	0.412
	Morning		**0.232**	**0.024**
	Evening	*NR1D1*	−0.042	0.660
	Morning		**0.249**	**0.010**
PSG parameters	Sleep efficiency		−0.127	0.301
	Sleep onset latency		0.045	0.615
	Sleep maintenance efficiency		−0.144	0.243
	REM sleep latency		0.055	0.534
	TST		0.037	0.675
	REM		−0.024	0.793
	N1 nREM		0.126	0.157
	N2 nREM		−0.116	0.193
	N3 nREM		−0.021	0.813
	nREM		0.010	0.912
	Arousal index		0.125	0.160
	AHI		0.159	0.071
	AHI in REM		0.134	0.134
	AHI in nREM		**0.184**	**0.039**
	Total number of desaturations		0.166	0.172
	Desaturation index		**0.179**	**0.041**
	Basal SpO_2_		−0.148	0.095
	Mean SpO_2_ during desaturations		−0.138	0.118
	Minimum SpO_2_		−0.107	0.256

*AHI* apnea-hypopnea index, *AM* subjective amplitude, *BMAL1* brain and muscle ARNT-like 1, *BMI* body mass index, *CLOCK* circadian locomotor output cycles kaput, *CRY1* cryptochrome 1, *CQ* Chronotype Questionnaire, *ESS* Epworth Sleepiness Scale, *ISI* Insomnia Severity Index, *MADRS* Montgomery-Åsberg Depression Rating Scale, *ME* morningness-eveningness, *NPAS2* neuronal PAS domain 2, *NR1D1* nuclear receptor subfamily 1 group D member 1, *nREM* non-rapid eye movement, *OSA* obstructive sleep apnea, *PER1* period 1, *REM* rapid eye movement, *SpO*_*2*_ oxygen saturation, *TST* total sleep time.

Bold values in the tables mark statistically significant data.

Among OSA participants, a multivariable linear regression was constructed for the MADRS score. The model explained 54.3% of the variance (*p* < 0.001) and included the following parameters: ISI score (*B* = 0.750, *p* < 0.001), AM score of CQ (*B* = 0.416, *p* = 0.007), and morning *PER1* gene expression (*B* = 4.310, *p* = 0.042). All parameters of the model are presented in Table 4.

**Table 4:** Linear regression model for MADRS score.

MADRS score				
Model	*R*^2^ = 0.543, *F* = 31.256, *p* < 0.001			
Parameter	*B*	*B* 95% CI	*t*	*p* value
Constant	−6.603	−11.935–(−1.272)	−2.465	**0.016**
ISI score	0.750	0.490–1.009	5.744	**<0.001**
AM score of CQ	0.416	0.114–0.717	2.744	**0.007**
morning PER1 gene expression	4.310	0.165–8.456	2.069	**0.042**

Excluded variables: ME score of CQ, ESS score, AHI in nREM, Desaturation Index, morning BMAL1 gene expression, morning CLOCK gene expression, morning CRY1 gene expression, morning NPAS2 gene expression, morning NR1D1 gene expression.

*AM* subjective amplitude, *BMAL1* brain and muscle ARNT-like 1, *CLOCK* circadian locomotor output cycles kaput, *CRY1* cryptochrome 1, *CQ* Chronotype Questionnaire, *ISI* Insomnia Severity Index, *MADRS* Montgomery-Åsberg Depression Rating Scale, *NPAS2* neuronal PAS domain 2, *NR1D1* nuclear receptor subfamily 1 group D member 1, *PER1* period 1.

Bold values in the tables mark statistically significant data.

## Discussion

The dysregulation of circadian gene expression in OSA has garnered significant attention in recent discussions, particularly in relation to the role of nocturnal intermittent hypoxia and the potential impact of disrupted circadian rhythms in OSA.

In this study, we demonstrate that expression of all examined circadian clock genes in OSA is upregulated in the morning compared to the evening. Additionally, we found a positive correlation between the severity of depressive symptoms (assessed with MADRS) and morning expression of circadian genes in the group of OSA patients. Finally, we found several prediction factors for the presence of greater severity of depression symptoms in OSA patients.

Our results suggest that disruption of circadian clock gene expression in OSA correlates with depressive symptoms. This observation is in line with the previous work regarding the association between depression and disruption of circadian rhythms. Partonen et al. demonstrated that sequence variations in three clock genes including *PER2*, *BMAL1*, and *NPAS2* may predispose to the development of winter depression [24]. Additionally, Bunney et al. examined post-mortem brain tissue comparing circadian gene expression in control subjects and MDD patients [25]. Results of the study showed a significant and marked loss in rhythmicity in the cyclic genes in the depression patients compared with controls [25]. What is more, Gouin et al. found that individuals with a history of depression had higher *CLOCK*, *PER1*, and *BMAL1* mRNA levels, compared to non-depressed participants [26]. That observation matches our results in terms of the correlation between elevated morning expression of clock genes in patients with OSA and greater severity of depressive symptoms.

To the best of our knowledge, this is the first study to provide evidence regarding disturbed clock gene expression in OSA and its involvement with the presence and severity of depressive symptoms. The multivariable regression model showed that *PER1* expression in the morning could be a positive prediction factor for more severe affective symptoms in OSA patients. What is more, greater amplitude of chronotype and insomnia severity also predicted an increase in the MADRS score in OSA patients, which indicates that patients with high diurnal variability of arousal as well as patients with more intense insomnia symptoms experience more severe depressive symptoms. The dysregulation in circadian rhythmicity, as well as sleep fragmentation in OSA, may lead to more liable chronotype and sleep disruption which could be a possible cause of affective disorders in OSA, however, the interaction is likely to be bidirectional and it is hard to establish a basis for unidirectional cause and effect relationship [27]. It has been previously described that higher amplitude in diurnal arousal may increase individual susceptibility to mood swings and affective disorders, which is also reflected in our data [22]. Our results may suggest that dysregulated expression of circadian clock genes and overall disruption of circadian rhythmicity in OSA may be a possible pathomechanism underlying the close connection between OSA and affective disorders. However, further investigation is required to provide a more precise evaluation of circadian gene dysregulation in both conditions.

The overnight disruption of circadian gene expression, observed in our study, may be mediated by nocturnal intermittent hypoxia (IH). Recent animal studies show that IH exposure results in changes in clock gene expression, especially in the lungs, where the hypoxemic impact is the most intense [28]. Studies with OSA patients provide evidence that HIF-1α as an oxygen-sensitive transcription factor contributes to the regulation of circadian rhythms [29]. It has been described that changes in oxygenation in rats may influence circadian rhythm disruptions in HIF-1α dependent manner [28]. Moreover, various studies present evidence suggesting that the elevated expression of clock genes, particularly *PER1*, in OSA patients may be restored to normal levels following the initiation of CPAP therapy [29]. On the other hand, the results from a larger study show, that CPAP treatment in OSA patients does not fully revert OSA-induced alterations in the expression of clock genes [30]. What is more, it has been also suggested that upregulated clock genes may predict the severity of OSA [31]. Additionally, some authors hypothesize that primary circadian dysregulation may contribute to the development of OSA [32].

The findings of this study hold important implications for both clinical practice and future research. Clinically, understanding the relationship between circadian gene dysregulation and depressive symptoms in OSA patients could guide the development of more targeted therapeutic interventions, such as chronotherapy or personalized CPAP treatment plans aimed at restoring normal circadian rhythms. In addition, these results suggest that routine screening for depressive symptoms in OSA patients, alongside assessments of circadian rhythm disruptions, may improve patient outcomes. Future research should build upon these findings by exploring the underlying mechanisms of circadian dysregulation in OSA and depression, potentially leading to novel treatment approaches that address both conditions simultaneously.

Several limitations of this study should be acknowledged. First, while the primary circadian clock is located in the brain’s suprachiasmatic nucleus, our investigation relied on peripheral blood leukocytes as a model for studying circadian rhythms in humans. Although these cells originate from peripheral tissues, their gene expression patterns are comparable to those observed in central tissues. Furthermore, circadian clock gene expression was measured at only two-time points, which may limit the detection of subtle interactions between chronotype and circadian genes. Another limitation is that depressive symptoms were assessed using a single questionnaire. It is also important to note that the study groups were not evenly matched, with the OSA group including individuals with varying degrees of OSA severity.

## Conclusion

OSA patients have dysregulated expression of circadian clock genes that are associated with the severity of depressive symptoms. Furthermore, expression of the *PER1* gene, with high diurnal variability of arousal and intensity of insomnia symptoms may play a role in predicting the development and severity of depressive symptoms in the group of OSA patients. Further research on a larger population is needed to investigate the complex pathomechanism of circadian disruption in OSA and its connection to affective disorders with special attention to the evaluation of possible cause and effect of otherwise bidirectional relationships between depressive symptomatology, chronotype predisposition, expression of circadian clock genes as well as results of OSA such as sleep fragmentation due to recurrent nocturnal arousals.

## Data Availability

Data will be made available upon reasonable request.
